# Prevalence of and risk factors for cystic echinococcosis among herding families in five provinces in western China: a cross-sectional study

**DOI:** 10.18632/oncotarget.21229

**Published:** 2017-09-23

**Authors:** Ruixia Yuan, Hairong Wu, Heng Zeng, Ping Liu, Quangang Xu, Lu Gao, Yin Li, Rendong Li, Duan Huang, Chuanhua Yu, Xiangdong Sun

**Affiliations:** ^1^ Department of Epidemiology and Biostatistics, School of Health Sciences, Wuhan University, Wuhan 430071, Hubei Province, China; ^2^ China Animal Health and Epidemiology Center, Qingdao 266032, Shandong Province, China; ^3^ College of Journalism and Communication, Guangxi University, Nanning 530004, Guangxi Province, China; ^4^ Institute of Geodesy and Geophysics, Chinese Academy of Sciences, Wuhan 430077, Hubei Province, China

**Keywords:** cystic echinococcosis, prevalence, risk factors, herding families, China’s western provinces

## Abstract

Echinococcosis is a severe zoonosis that endangers the health of herdsmen in China’s western provinces. This study aimed to examine the prevalence of this disease and identify potential factors associated with human echinococcosis among herding families.

A cross-sectional study was conducted in five provinces in western China from May 1, 2016 to November 30, 2016, and 1500 herding families participated in the study. A total of 1211 completed questionnaires were analyzed. The prevalence of Cystic echinococcosis (CE) among surveyed herding families was 1.55%. The results of multivariate analysis revealed that the sheep immunization (OR=0.35, 95%CI 0.21-0.58), being concerned about family members echinococcosis (OR=0.49, 95%CI 0.28-0.84) were protective factors, while allowing dogs to roam free (OR=3.17, 95%CI 1.89-5.31), feeding dogs with viscera (OR=3.04, 95%CI 1.83, 5.03), slaughter at home (OR=3.53, 95%CI 2.04-6.12), drinking non-boiled water (OR=2.15, 95%CI 1.28-3.63), eating raw vegetables (OR=1.87, 95%CI 1.13-3.10), not washing hands before meals (OR=3.08, 95%CI 1.68-5.65), and often seeing stray dogs (OR=2.60 95%CI 1.38-4.91) and wild animals (OR=1.92, 95%CI 1.17-3.14) near habitations were more associated with increased risk of infection.

Immunizing sheep, appropriately managing domestic and stray dogs, and improving living environments and behavioral factors may help to reduce the risk of human echinococcosis in western China.

## INTRODUCTION

Echinococcosis, also called hydatid disease, is a zoonotic parasitic disease associated with economic losses in the livestock industry and serious human health risks [1]. There are over 1 million people infected with echinococcosis worldwide at any given time [2]. Cystic echinococcosis (CE) caused by Echinococcus granulosus is the most common form identified in humans in the global context [3]. According to data from the WHO, CE was found to be widely distributed in most pastoral and rangeland areas worldwide, and associated with a burden.

Human echinococcosis has a long incubation period and complicated transmission routes [4]. Common definitive hosts include dogs, wolves, fox and other carnivorous animals [5]. Large numbers of adult hydatid parasites are often identified in the small intestine of definitive host animals. Gravid proglottides, or eggs produced by adult parasites, are released from the body via the host’s droppings. Intermediate hosts, which include herbivorous and omnivorous animals [6], eat eggs or proglottid from polluted soil, water, pasture, etc. Humans are accidental intermediate hosts and, similar to other intermediate hosts, acquire infection via the fecal-oral route; however, humans do not participate in the transmission cycle [7].

A recent meta-analysis [8] showed that present studies on CE have identified the following predictive factors: source of infection, such as “dog ownership”; route of transmission, such as food- and water-borne transmission; and socio-cultural, such as age, income, gender and education level. A systematic review [9] summarized the epidemiological factors associated with increased risk of echinococcosis infection in dogs and intermediate hosts and reported that being fed raw viscera, lacking anthelmintic treatment, and having owners that lack health education and were impoverished were risk factors. Khazaei et al. [10] analyzed the characteristics of patients with hydatid cysts and suggested that it is necessary to identify infection sources among people at high risk.

China has the highest prevalence rate of human echinococcosis worldwide [11, 12]. The major epidemic areas are provinces in western China and the border areas of the Qinghai-Tibet Plateau [13]. An analysis of the endemic status of echinococcosis in China showed that 10 790 cases of echinococcosis were reported in China from 2004 to 2008, 87.3% of which were identified in patients from the Xinjiang Uygur Autonomous Region, Tibet Autonomous Region, and Gansu, Sichuan and Qinghai Provinces. [14]

Several studies have evaluated echinococcosis risk factors in China in recent years. Bai Y et al. [15] surveyed 451 Tibetans students living in rural Tianzhou County in Gansu Province and suggested that age, gender and hunting status had an effect on the disease infection. Schantz PM et al. [16] surveyed 3703 volunteers in Qinghai Province from June 1997 to June 1998, and the results of the multivariate analysis suggested that potential risk factors included livestock ownership, age > 25 years, female gender, herding occupation and nomadic status. Li TY et al. [17] conducted a study in Shiqu County, Sichuan Province, which has been reported to have the highest human echinococcosis prevalence in the world [18], and found that risk factors included age, gender, dog ownership and sources of drinking water. However, thus far, these studies have had some limitations that should be considered, as most of the studies occurred in an individual city or county, and none of the studies analyzed the influence of intervention measures on human echinococcosis, which, at present, mainly include sheep immunization, dog deworming and health education in China [19].

Thus, the China Animal Health and Epidemiology Center (CAHEC) of Ministry of Agriculture of the People’s Republic of China (MoA) conducted this study to obtain a greater understanding of the prevalence of human echinococcosis in western China, identify the factors associated with the disease infection and evaluate the current measures.

## RESULTS

### Participant characteristics

The characteristics of the 1211 herding families are presented in Table 1. The average number of family members was 4.8±1.6, which was higher than the national average (3.02, 2015) [20]. More than 90% of the investigated families were dog owners, and the average number of dogs owned was 1.2. A total of 90 patients with CE were identified which were distributed among 90 families.

**Table 1:** Demographic characteristics of herding families surveyed

Type	N (%)	Mean±SD
Male	1022 (84.4)	
Age (year)		46.2±11.8
Below the junior middle school education	1073 (88.6)	
Family population		4.8±1.6
Religions		
Buddhism	805 (66.5)	
Islam	243 (20.1)	
Others	163(13.5)	
Languages most often spoken		
Chinese	275(22.7)	
Tibetan language	770 (63.6)	
Others	166 (13.7)	
Raising livestock	1211 (100)	
Cattle restock		19.5±22.4
Sheep restock		135.1±171.5
Households owned dogs	1114 (91.9)	
The number of domestic dogs		1.2±0.9
The reasons for keeping dogs		
Help graze	784 (64.7)	
As a pet	603 (49.8)	
Housesitting	35 (2.9)	
Others	712 (58.8)	
Producing activity and living environment		
The sheep immunization	413(34.05)	
Domestic dogs deworming	537(44.30)	
Dog free to roam	819(67.52)	
Slaughter at home	547(45.09)	
Feed dogs with viscera	765(63.07)	
Drink unboiled water	892(73.54)	
Eat raw vegetables	518(42.70)	
Do not wash hands before meals	1011(83.35)	
Often see stray dogs near habitations	783(64.55)	
Often see wild animals near habitations	416(34.30)	
Being concerned about the livestock echinococcosis	495(40.81)	
Being concerned about family members echinococcosis	494(40.73)	

### Prevalence of human echinococcosis

Ninety patients who had been diagnosed with human echinococcosis were identified among the surveyed families. When the number of households surveyed × average household size (5813) was used as the denominator value, the average CE prevalence was 1.55%. The prevalence rates of CE in different provinces are shown in Table 2.

**Table 2:** The prevalence of CE in different province

Provinces and regions	The no. of patients	The no. of population	Prevalence (%)	*χ2*	*P*
Xinjiang Uygur Autonomous Region	14	1365	1.03		
Tibet Autonomous Region	25	996	2.51	11.53	0.020
Gansu Province	11	1123	0.98		
Sichuan Province	21	1258	1.67		
Qinghai Province	19	1072	1.77		
Total	90	5813	1.55		

The results of the chi-square test suggested that the presence of significant between province difference in the prevalence of CE (p<0.05). Tibet had the highest prevalence, which was 2.51%.

### Single factor analysis of CE infection

The presence of CE patients in a family was used as the dependent variable, and the influences of 11 predictor variables, including “immunizing sheep”, “feeding dogs with viscera”, and “allowing dogs to roam free”, et. Al., were analyzed using single factor logistic regression analysis. All of these variables were binary variables. The results are shown in Table 3.

**Table 3:** Factors related to having CE patients in a family: single factor analysis

Variables	β	*Wald*	*P*	OR	95% CI	
					Lower	Upper
The sheep immunization	-1.05	16.59	0.000	0.35	0.21	0.58
Domestic dogs deworming	-0.90	0.11	0.739	0.91	0.54	1.55
Allowing dogs to roam free	1.16	19.15	0.000	3.18	1.89	5.33
Slaughter at home	1.12	18.44	0.000	3.02	1.83	5.01
Feeding dogs with viscera	1.24	18.75	0.000	3.46	1.97	6.07
Drinking non-boiled water	0.78	8.31	0.004	2.18	1.28	3.71
Eating raw vegetables	0.62	5.81	0.016	1.86	1.12	3.08
Not washing hands before meals	1.11	12.71	0.000	3.04	1.65	5.59
Often seeing stray dogs near habitations	0.97	8.87	0.003	2.63	1.39	4.98
Often seeing wild animals near habitations	0.65	6.59	0.010	1.91	1.16	3.12
Being concerned about family members echinococcosis	-0.73	6.71	0.010	0.48	0.28	0.84

### Multiple factor analysis of CE infection

Variables identified as statistically significantly in the single factor analysis were included in the multifactor logistic regression analysis. The analysis results, which are presented in Table 4, showed that the sheep immunization and being concerned about family members echinococcosis were associated with reduced the risk of infection, while two dog-related factors (allowing dogs to roam free, feeding dogs with viscera), four living habit-related factors (slaughter at home, drinking non-boiled water, eating raw vegetables, not washing hands before meals), and two living condition-related factors (often seeing stray dogs or wild animals near habitations) were significantly associated (*p<*0.05) with increased risk of human echinococcosis.

**Table 4:** Factors related to having CE patients in a family: multiple factor analysis

Variables	β	*Wald*	*P*	OR	95% CI	
					Lower	Upper
The sheep immunization	-1.05	16.59	0.000	0.35	0.21	0.58
Allowing dogs to roam free	1.15	19.09	0.000	3.17	1.89	5.31
Feeding dogs with viscera	1.11	18.60	0.000	3.04	1.83	5.03
Slaughter at home	1.26	20.25	0.000	3.53	2.04	6.12
Drinking non-boiled water	0.77	8.20	0.004	2.15	1.28	3.63
Eat raw vegetables	0.63	5.95	0.015	1.87	1.13	3.10
Not washing hands before meals	1.12	13.16	0.000	3.08	1.68	5.65
Often seeing stray dogs near Habitations	0.96	8.76	0.003	2.60	1.38	4.91
Often seeing wild animals near habitations	0.65	6.69	0.010	1.92	1.17	3.14
Being concerned about family members echinococcosis	-0.72	6.62	0.010	0.49	0.28	0.84
Constant	-4.87	126.53	0.000	0.08	-	-

## DISCUSSION

According to the WHO, incidence of human CE may exceed 50/100 000 in endemic regions. The results of a national survey regarding the hydatid disease epidemic that was conducted in 2012 showed that the average prevalence of human echinococcosis was 0.24% in the 263 counties in western China. We identified a higher prevalence in the study (1.55%). We identified a higher prevalence in the study (1.55%). Echinococcosis is an often neglected disease with a long incubation period [21]. This prevalence is likely to increase because more patients may be detected as awareness of the harms associated with echinococcosis increases among herdsmen and relevant national health support policy is implemented.

Dogs are the most common definitive host, and dogs have been identified as the primary source of human CE infection [22]. One previous study [23] showed that keeping dogs was associated with increased risk of human echinococcosis.

In our study, the results of the multiple regression result showed that “allowing dogs to roam free”, which may artificially accelerate the transmission cycle of echinococcosis, may increase the risk of infection. For example, a recent study showed that [24] the dog seropositivity rate was as high as 64.56% in Qinghai Province. Free dogs infected with adult hydatid parasites have been found to defecate openly, resulting in the dissemination of hydatid eggs into the surrounding areas. In addition, the main purpose reported for keeping dogs by herding families in our analysis to help graze according to our survey. For example, dogs stay with sheep during the grazing process. Wool may be contaminated with hydatid eggs from dog feces. Herders come into close contact with eggs during milking, shearing and other production activities and are likely to become patients.

“Feeding dogs with viscera” was also associated with increased likelihood of human infection. Surveys [25, 26] previously performed in these areas have shown that the seropositivity rate of cattle and sheep ranged from 5% to 30%. Offal from those cattle and sheep may be fed to dogs. Hydatid eggs may be consumed by dogs and gradually develop into adult parasites in the small intestine. Thus, a new cycle of echinococcosis transmission begins.

Previous studies [27, 28] have identified slaughterhouses as possible influencing echinococcus risk. This is consistent with our findings. Livestock has been found to serve as an intermediate host [29, 30]. We estimated that “slaughtering at home” may increase the risk of human echinococcosis because offal from sick animals are usually fed to domestic or stray dogs by herdsmen rather than safely disposing of these viscera during slaughter, directly contributing to increased risk of human echinococcus.

As previously mentioned, humans become “victims” because of consuming proglottid or eggs in contaminated food or water. The results of this study showed that some factors related to the living habits, such as “’not washing hands before meals”, “drinking non-boiled water” and “eating raw vegetables” may contribute to increased risk, results that were consistent with those of previous studies [16, 31, 32].

In pastoral areas, herdsmen often drink non-boiled water from rivers or lakes, and animals also drink from the same water sources while grazing. Eating raw vegetables or other foods is also very common. Tsampa, which is often consumed with the fingers, is the important component of the diet of herding families. However, members of these families are often unable to wash their hands before eating due to a lack of water or being busy with production activities. Echinococcosis eggs in the external environment can go enter the body through these bad habits.

The following two environmental-related factors were identified: often seeing stray dogs and often seeing wild animals near habitations. These variables were included in analysis, and both of these variables were identified as potential risk factors for infection. This finding was similar to the results of a previous study that found that living environment has an effect on human echinococcosis [32]. Another study found that similar to dogs, common wildlife species such as wolves and foxes may act as disease vectors [29]. This finding suggested that governing stray dogs and keeping habitations away from stray dogs and wild animals may reduce the risk of echinococcosis.

To determine whether prevention and control measures have positively impacted the occurrence of echinococcosis infection among herdsmen, we evaluated and attempted to analyze the etiology of infection. Although 44.30% of the surveyed herding families could provide dogs with anti-parasitic agents, the deworming of domestic dogs had no effect on human infection in our study. Similar to Gerardo AJ’s study [33], the effect of this variable was not significant. This finding may be related to the difficulties in completely deworming identified during our field observations and interviews. First, it is difficult to deworm drugs at the recommended frequency (every dog, every month). Additionally, being busy with production activities or psychological indifferent made feeding the deworming tablets according to recommendations more difficult. Second, safe disposal of feces from dogs after deworming was also a challenge. When feces are not deeply buried or burned, hydatid eggs may survive.

Osbom PJ’s study showed that [34] resistance to immunization among sheep spines ball larva may effectively resist various fine grained spines ball larva and reduce the incidence of sheep echinococcosis infection. Control of sheep infection is beneficial to cut off the transmission chain of cystic echinococcosis [30]. The results of the regression analysis showed that herding families who immunized their sheep were 0.35 times less likely to develop echinococcosis than those who did not immunize their sheep. The “national animal disease compulsory immunization program 2016” [35] issued by the Chinese Ministry of Agriculture stated that the immunization strategy used for echinococcosis should be independently selected by animal husbandry and veterinary departments and other relevant departments at the provincial level according to the actual needs in highly endemic areas. This recommendation suggests that it may be necessary to revise immunization processes in these areas to ensure the prevention and control of echinococcus in the future.

Being concerned about echinococcosis in livestock or family members served as a reflection of the herdsmen’s health education regarding the prevention and control of echinococcosis. Craig PS’s study [36] analyzed echinococcosis interventions in various countries and regions around the world from 1986 to 2002 and found that health education had little effect on the risk of echinococcosis transmission. Similar results were identified in our research. Further study of the effect of health education on human echinococcosis in the evaluated western provinces and regions is the next objective of this research group.

Our study has several strengths. China has the largest human hydatid disease epidemic, especially in the western provinces and autonomous regions. Carrying out epidemiological surveys that includes the populated areas instead of just individual provinces will be necessary to obtain a better understanding of this disease. The 15 counties surveyed in this study were distributed in five provinces in western China. Using the data obtained from these surveys, we briefly described the prevalence of human echinococcosis in five provinces, including the Tibet Autonomous Region, which had not been assessed previously. In addition to evaluating host factors, living habits and living environment, existing prevention and control measures were also analyzed. Because of these advantages, our results could be utilized to support the prevention and control of hydatid disease in China according to the local conditions.

This study had two limitations. First, individual characteristics that may have effected infection, such as gender and level of education, were not included in the models. These variables were excluded because our analysis focused on the herding family unit and, specifically, the life styles, living environments and prevention and control measures there in. Furthermore, we included a limited number of affected families, and an analysis of whether different provinces have different risk factors could not be performed in this study. More in-depth investigations and comparative analyses will be carried out in 2017.

In conclusion, the prevention and control of echinococcosis is a priority in western China. Immunizing sheep and appropriately managing domestic and stray dogs should serve aseffective measures for controlling CE at present. Efforts to improve living environments and behaviors should consider the natural conditions, characteristics of production and the lives of herdsmen’s families. Further studies are needed to clarify the importance of health education.

## MATERIALS AND METHODS

### Ethics statement

The research protocol was approved by the CAHEC of MoA (Nong Fa [2016] No. 11). The questionnaire survey received ethics approval from the Division of Epidemiology Survey within CAHEC [37]. All participants must sign a written informed consent when they were informed about the purpose and procedures of the study. In case of anyone not understanding Chinese, investigators from the local veterinary station explained in minority language. There were no animal samples taken as part of our study.

### Participants

For the number of echinococcosis families in western provinces and regions is unclear at present, π=0.5, allowable error δ=0.03, ɑ= 0.05. 1067 is the smallest unit of observation in the calculation of sample size. In our study, 1500 herding families were screened in Xinjiang Uygur Autonomous Region, Tibet Autonomous Region, and Gansu, Sichuan and Qinghai Provinces. Considering the vast and sparse population in these areas, a combination of probability and convenience sampling were used. The full process of sample selection was as follows: three animal husbandry counties were selected by Animal Diseases Control and Prevention Centers in each of the 5 provinces according to the probability of investigative work; then, 100 families were randomly selected in each county by computer. According to the information provided by the county veterinary department, the SPSS software was used to achieve a completely random. The steps were: data - select cases - random sample of cases and the definition sample size = 100.

### Study area

**Figure 1:**
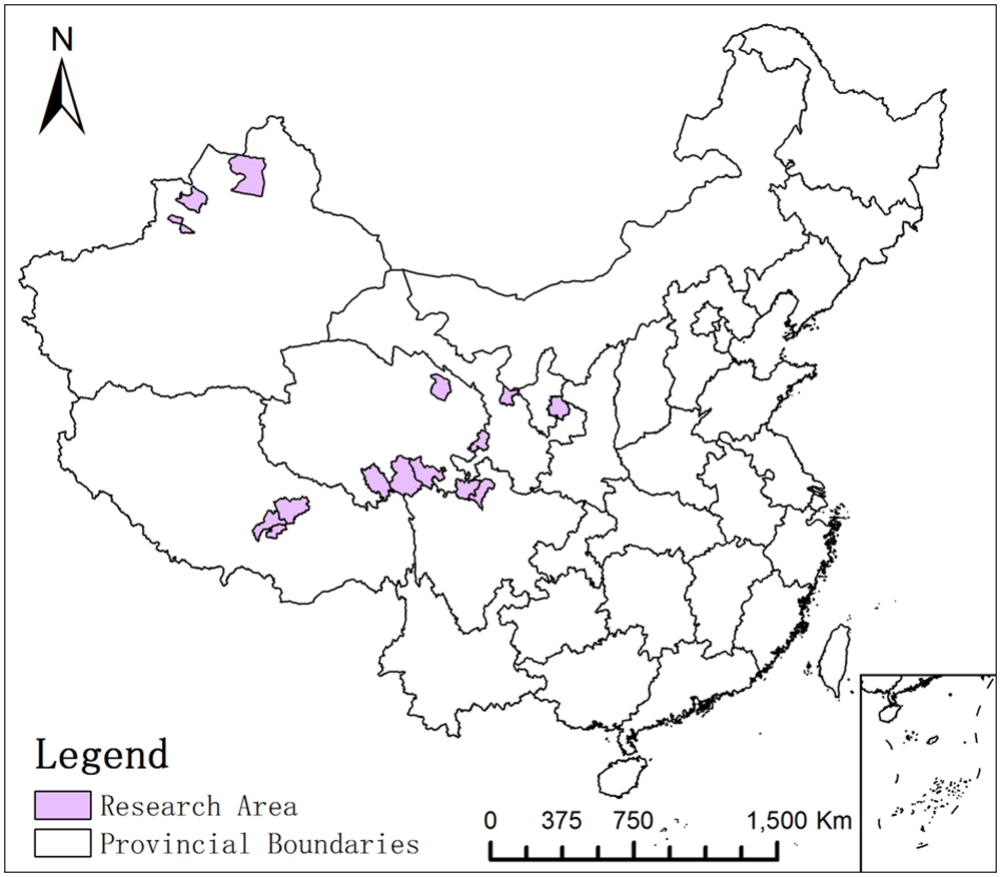
15 counties conducted in the research

The research was conducted in 15 counties (Figure 1. Vector map filecame from Resources and Environment Science Data Center, Chinese Academy of Sciences and was performed with ArcGIS10.2 software). The terrain of these areas is complex, especially in the Tibet, Sichuan and Qinghai areas, and the average altitude in the surveyed counties was above 3000 meters. Animal husbandry is the main form of agricultural production in these areas.

### Method and content

The survey was carried out from May 1, 2016 to November 31, 2016. Data were collected using questionnaires that included the following 5 parts: sociodemographic characteristics, echinococcosis infection in family members, livestock breeding situation, production activity and living environment. The questionnaire was used in Chinese. The staffs from the county veterinary department were responsible for the translation of minority languages and Chinese between the investigators and the subjects when they were not familiar with Chinese. The head of the household, which was defined as the person who in charge of the household and any dependents [38], was responsible for completing the questionnaire. Case confirmed was based on the results of the investigation of the family’s active medical treatment or the health examination that has been carried out in these areas. Respondents were asked to provide relevant diagnostic material to confirm the presence of human echinococcosis among family members. The diagnostic materials include two types: the diagnosis certificate issued by the hospital; the results of the imaging examination. When the answer of respondent is “yes” for the question of “whether or not there is a family member (including oneself) suffering from hydatid disease”, we ask one of the two. The design of the survey questionnaire and training of investigators were completed by the CAHEC.

### Statistical analysis

Overall, 1213 completed questionnaires were collected and the completion rate was 80.87%. Two families located in Sichuan Province with alveolar echinococcosis (AE) patients were excluded. AE was caused by *Echinococcus multilocularis*. It is well-known that life-cycles of Echinococcus granulosus and E. multilocularis are quite different [39]. As a result, 1211 were analyzed, Statistical analysis was performed using SPSS 20.0. The demographic data for the heads of household and characteristics herding families were analyzed by using descriptive statistics and chi-squared test. Binary logistic regression with forced entry was performed to explore the risk factors for human echinococcosis. In all statistical analyses, *p*<0.05 was considered statistically significant.
